# Anisotropic Multi-channel Collagen Gel (MCCG) Guides the Growth Direction of the Neurite-like Processes of PC12 Cells

**DOI:** 10.1038/s41598-018-32156-0

**Published:** 2018-09-17

**Authors:** Isabel Koh, Kazuya Furusawa, Hisashi Haga

**Affiliations:** 1 Graduate School of Life Science, Hokkaido University, Kita-ku Kita 10 Nishi 8, Sapporo, 060-0810 Hokkaido Japan; 2 Faculty of Advanced Life Science, Hokkaido University, Kita-ku, Kita 10 Nishi 8, Sapporo, Hokkaido 060-0810 Japan; 3 Global Station for Soft Matter, Global Institution for Collaborative Research and Education, Hokkaido University, Sapporo, Hokkaido Japan; 4 Department of Environmental and Food Sciences, Fukui University of Technology, Gakuen 3-6-1, Fukui, Fukui, 910-8505 Japan

## Abstract

Hydrogels made of various materials using a variety of methods have been extensively studied for use in tissue engineering, and collagen is one of the most common material used for its biocompatibility due to it being a major component of the extracellular matrix (ECM). Furthermore, the alignment of collagen fibres has been shown to direct the growth of neurites, an important criterion for engineering nervous tissues. The Multi-channel Collagen Gel (MCCG) has collagen fibres aligned circumferentially around the channel structures of the gel, and we predicted that the MCCG could guide the growth direction of neurites. In this study, we showed that the growth pathway of the neurite-like processes of PC12 cells were guided in MCCG but not in normal collagen gel (COL). The gelation of collagen gels are known to be affected by ionic concentrations, and hence we also investigated the effects of different concentrations of NaCl on the properties of MCCG. We found that, despite differences in channel density, spacing between channels, and degree of collagen fibre alignment, all MCCGs had similar guiding properties on the growth of neurites. Therefore, we believe that anisotropic MCCG could be a useful biomaterial for neural tissue engineering in the future.

## Introduction

The field of 3D tissue engineering that encompasses the study of a wide range of materials and methods aims to create models that mimic the native tissue, either to provide researchers a more representative tool for *in vitro* studies, or for potential clinical applications. Accordingly, many review articles have been written on the subject^1–4^, covering the desired properties of engineered tissues, the various materials and fabrication techniques to make these, as well as their respective advantages and disadvantages.

The basic premise of the field is to construct a scaffold, which aims to mimic the extracellular matrix (ECM) in which cells are either encapsulated or directly seeded onto. For this to be successful, the scaffold should: (i) be biocompatible and not cause cytotoxicity, or severe inflammation if implanted in the body, (ii) allow good cell infiltration, attachment, and survivability, (iii) be biodegradable, and (iv) possess mechanical and chemical properties similar to the native tissue^5,6^. Particularly for neural tissue engineering, however, the scaffolds should also have the capability to guide the growth direction of neurons to form a connecting network. This is often achieved by aligning the fibres of the polymers being used to construct the scaffold, and various polymers have been shown in several studies to provide directional guidance to extending neurites when aligned, including collagen^7–9^, fibrin^10^, poly(ε-caprolactone) (PCL)^11^, and polylactic acid (PLA)^12^.

**Figure 1:**
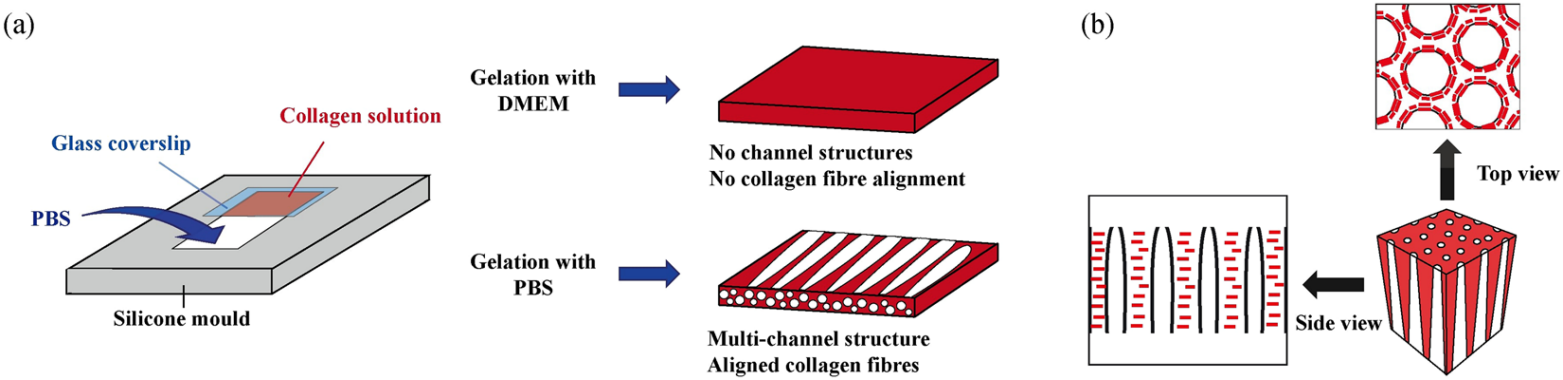
(**a**) Multi-channel Collagen Gel (MCCG) is prepared simply by adding a phosphate buffer solution (PBS) to neutralize the acidic collagen solution. This results in a collagen gel with a multi-channel structure and aligned collagen fibres. On the other hand, normal collagen gel (COL) is prepared using DMEM in place of PBS, resulting in a collagen gel with no channel structures and no collagen fibre alignment. (**b**) When viewed from the top, the fibres appear aligned parallel to the edges of the channel lumen, and viewed from the side, the fibres are parallel to one another but perpendicular to the channel structure.

Of these, collagen is often used in scaffold construction as it is a major component of the ECM, and can satisfy many of the above-mentioned criteria. We have developed a collagen hydrogel with a high density of channel structures (Multi-channel Collagen Gel, MCCG)^13^, prepared simply by neutralising acidic collagen solution with a gelating phosphate buffer solution (PBS). On the other hand, collagen hydrogels with no channel structures (COL) are prepared with DMEM as the gelating reagent (Fig. 1a). Conventional methods for preparing COL via the neutralization of collagen solution include using NaOH-KH_2_PO_4_ or Ham’s F-12 as the gelating reagent, and are widely thought to proceed by the nucleation and growth-type phase separation^14,15^, whereas the formation mechanism for MCCG could be attributed to proceed by spinodal decomposition^13^.

In the nucleation and growth phase separation process, nuclei of collagen fibrils are formed in the early stages of the neutralization, and as more and more collagen molecules are arrested by the nuclei, the nuclei grow in size laterally and longitudinally and form collagen fibres. The collagen fibres eventually connect to form a single three-dimensional collagen gel network. In spinodal decomposition, the neutralization initiates the formation of alternating polymer-rich and polymer-poor regions, and as gelation proceeds, collagen molecules continue to flow towards the polymer-rich regions, thus creating the collagen matrix and channel lumen regions of the MCCG.

From the birefringence property shown by the MCCG under polarized light, it was deduced that the collagen fibrils in MCCG were oriented circumferentially around the lumen of the channels^13,16^. In other words, when the MCCG is viewed from the top, the fibres appear aligned parallel to the edges of the channel lumen, and viewed from the side, the fibres are parallel to one another but perpendicular to the channel structure (Fig. 1b). This alignment may be attributed to the large area of interfaces in the MCCG due to the presence of a high number of channels, leading to high interfacial free energy. In order to minimize this free energy, the collagen fibrils adopt a conformation of aligning parallel instead of perpendicular to the channel surface, as this reduces the surface area and hence surface free energy of the system.

We postulated that this alignment of collagen fibres may play a role in guiding the growth pathway of extending neurites. In this study, using rat adrenal medulla pheochromocytoma PC12 cells which extend neurite-like processes when treated with nerve growth factor (NGF) as model nerve cells, we examined the straightness and angle at which the processes extend from the channels in which they were seeded. We first compared cells seeded in MCCG with those seeded in normal collagen gel with no fibre alignment (COL). To create channel structures in COL to enable the seeding of cells, gold wires were placed in the collagen solution before gelation.

However, we should also take into consideration the fact that the channel structures may also have a role in guiding the neurite-like processes. While the collagen fibres give the MCCG an anisotropic property, the channel structures lend it an isotropic feature, and both anisotropy and isotropy are known to affect neuronal growth^17^.

As the number of channels we could mimic in COL were limited to four due to the method of gel preparation, we compared MCCGs prepared with different concentrations of sodium chloride (NaCl), which were previously shown to affect the phase behaviour of collagen solution, and subsequently the number and diameter of channels in the MCCG^18^. However, in that study, only the top and bottom 300 µm of the MCCG was studied, and the differences appear to plateau towards the inner regions of the gel. Additionally, changes in ionic strength has also been reported to affect the rate of collagen gel formation^14^, and we hypothesized that this may also have an effect on the degree of collagen fibre alignment of MCCGs. Thus, the second aim of this study was to characterize the gels at increasing depths and the alignment of collagen fibres, and to investigate the relationship between these characteristics and neurite growth direction in MCCGs with differing properties.

## Materials and Methods

### Cell Culture

PC12 cells were obtained from Riken Cell Bank (Tsukuba, Japan). Cells were cultured on dishes coated with 0.3 mg/mL Cellmatrix Type I-A (Nitta Gelatin Inc.) in low glucose DMEM (Wako) supplemented with 10% fetal bovine serum (Biowest), 10% horse serum (Biowest), and 1% penicillin-streptomycin. TrypLE Express Enzyme (Gibco) was used to detach cells. Differentiation medium comprised growth medium with 100 ng/ml mouse NGF 7S (Alomone Labs) added to it.

### Preparation of Multi-channel Collagen Gel (MCCG) and Normal Collagen Gel (COL)

**Figure 2:**
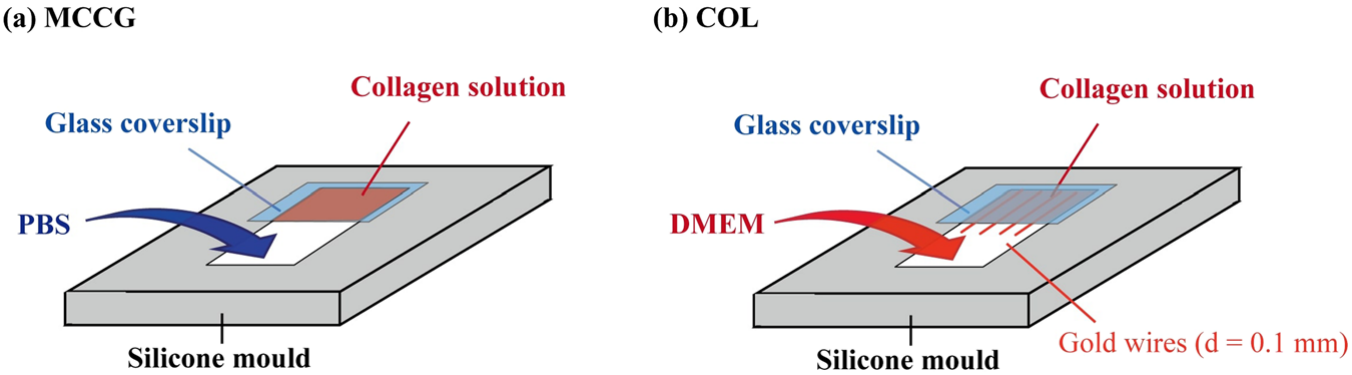
Method of gel preparation. (**a**) MCCG is prepared using PBS solution, whereas (**b**) COL is prepared using DMEM with gold wires placed in the collagen solution to imitate channel structures.

To prepare MCCG, a 1 mm-thick silicone mould and an 8 mm × 8 mm glass coverslip were used to create a chamber 5 mm in length and 6 mm in width in a culture dish, and 5 mg/ml AteloCell IPC-50 collagen solution (KOKEN) was pipetted into the chamber space (Fig. 2a). A phosphate buffer solution (gelation PBS; 20 mM Na_2_HPO_4_, 13 mM KH_2_PO_4_) was then slowly and carefully added to the open side of the chamber to initiate gelation, before filling the culture dish to cover the whole setup after about 1 min.

To prepare MCCGs with different properties, 100 mM NaCl stock solution was prepared and added to both the collagen solution and the gelation PBS to reach a concentration of 0 mM, 1 mM, 2 mM, and 3 mM NaCl.

To prepare COL with mimicked channel structures to enable cells to be seeded in the gel, the same chamber as described above was set up, and gold wires of 0.1 mm (Nilaco Corporation) placed in the collagen solution before adding growth medium to initiate gelation (Fig. 2b).

The gels were left at room temperature (20 °C) overnight for the collagen solution to gelate completely. The samples were then washed twice with PBS (−) (137 mM NaCl, 1.5 mM KH_2_PO_4_, 8.4 mM Na_2_HPO_4_, and, 2.3 mM KCl pH 7.4), and the dish filled with growth medium after the last wash. The glass coverslips, gold wires, and silicone moulds were removed, and to ensure that the channels are open to enable cell seeding, about 1 mm was cut off on both ends of the gel (the “top” and “bottom”). The samples were then incubated with culture medium at cell culture conditions (humidified incubator at 37 °C, 5% CO_2_) for at least 30 min before seeding.

To seed cells in the channels of the gels, 1 × 10^6^ cells/gel were added the dish, and the medium pipetted up and down 20 times near the channel opening (10 times on each side) to ensure as many cells as possible enter the channels. The samples were then incubated on a shaker at cell culture conditions overnight. The next day, the samples were detached from the culture dish and transferred to a 12-well plate with differentiation medium. Medium was changed every day thereafter.

### Extending Angle and Straightness of Neurite-like Processes

At the end of the culture period of 7 days, the samples were washed twice with PBS (−) before fixing with 4% paraformaldehyde overnight at 4 °C. After fixation, the samples were blocked with 1% BSA for 1 hr, and immune-labelled with COL1A1 (D-13) goat polyclonal IgG (Santa Cruz Biotechnology) diluted 200-fold in 1% BSA overnight 4 °C. Then, the samples were permeabilized with 0.5% Triton X-100 for 1 hr, and blocked with 1% BSA for 1 hr. Collagen was stained with Alexa Fluor 633 rabbit anti-goat IgG (Life Technologies) diluted 400-fold, nuclei stained with SYTOX Blue (Life Technologies) diluted 400-fold, and F-actin stained with 1 unit/ml Alexa Fluor 488 phalloidin (Life Technologies) in 1% BSA with 0.1% Triton X-100 for 6 hr. All steps were carried out at room temperature unless otherwise stated, and the samples were washed three times with PBS (−) between each step.

**Figure 3:**
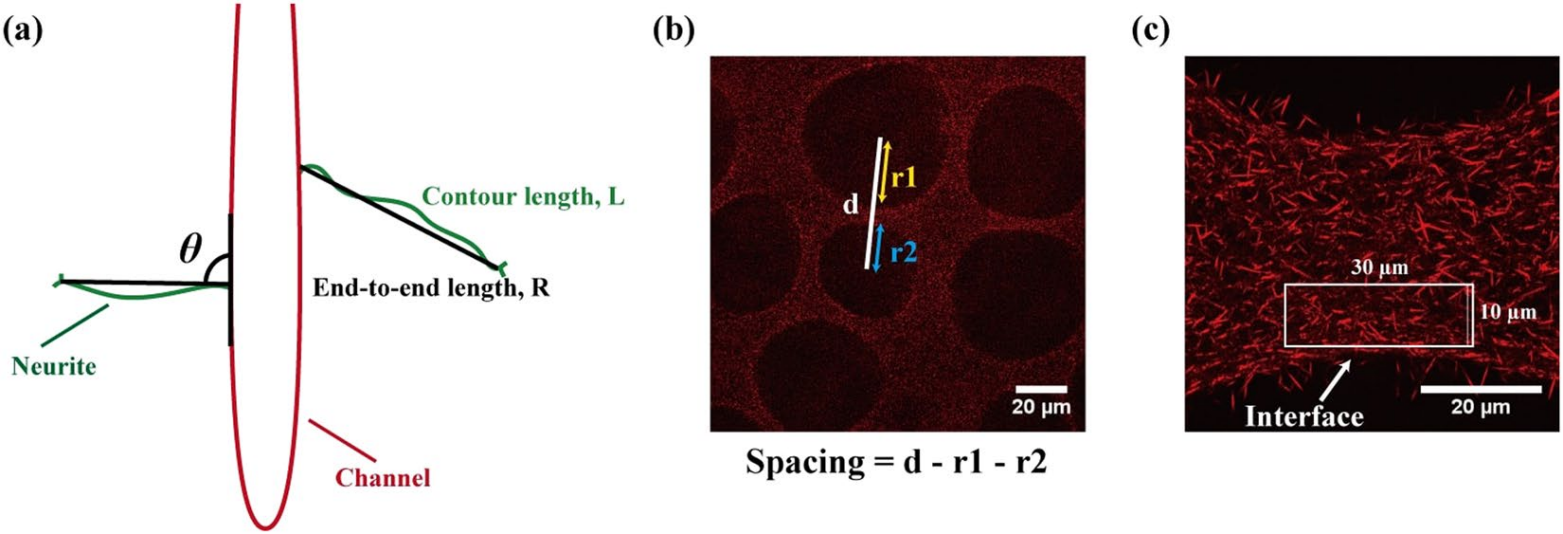
Analysis methods. (**a**) The angle at which the neurite-like processes of PC12 cells extend from the channel is measured as the angle from the growth cone to the channel structure, and the straightness of their growth is calculated as the ratio of contour length, L to end-to-end length, R of the process. (**b**) The spacing between two adjacent channels is measured by subtracting the radii of the two channels from the distance between the centres of mass of the channels. (**c**) The area on which FFT analysis is performed is a 30 µm × 10 µm window cropped from near the interface between the channel and collagen matrix.

To compare the angles at which the neurite-like processes extend into the collagen matrix relative to the channels, and their straightness, confocal laser scanning microscope (CLSM, Leica TCS-SP5) side view images were obtained as follows: 10x objective lens was used with a zoom factor of 4.0, and images were taken at 10 µm-volume stacks with a step size of 1.5 µm from z = 0 to z = 60 µm, with z = 0 µm being the position just above the glass coverslip. Images were obtained near the top half of the gels where the channels of MCCG are narrower, as the cells were more concentrated in this region due to the channels being narrower and closer to the inlet. Three sets of images were taken for each sample with *n* = 3 for MCCG vs. COL experiments, and two sets of images for each sample with *n* = 6 for the experiments with MCCGs of different ionic strengths. The angles at which the neurite-like processes extend out of the channels into the gel matrix region relative to the channel structure (θ), the contour length of the process (L), and its end-to-end length (R) were measured using ImageJ (Fig. 3a). Only processes whose entire length from channel surface to growth cone can be observed were measured. The straightness of the processes was calculated as the ratio of L/R, with a ratio of 1 indicating that the processes are completely straight, and the higher the ratio, the less straight the process is.

### Characterisation of MCCG

To characterize the morphological properties of MCCGs, confocal reflectance microscopy (CRM, Leica TCS-SP5) was used. MCCGs were prepared as above without seeding cells in them, and then fixed with 4% paraformaldehyde overnight at 4 °C. After washing 3 times with PBS (−), the gels were cut into 2 mm sections and flipped on its side to enable top view images to be obtained. For these experiments, 2 mm-thick silicone moulds were used instead of 1 mm-thick moulds for ease of cutting and handling of samples, and to obtain images where the channels are not distorted or damaged by cutting.

To determine the number of channels, diameter of channels, total area of channels, and the spacing between channels of MCCGs prepared with different NaCl concentrations, top view images were obtained as follows: 5x objective lens was used with a zoom factor of 1.7, and images were taken at 10 µm-volume stacks with a step size of 4.99 µm. Two images were taken for each sample, and *n* = 6. For the number of channels and total area of channels, all channels were counted and measured, including incomplete channels around the edges, whereas only whole channels were taken into account when measuring the diameters. The spacing between channels was calculated by measuring the distance between the centres of mass of two channels and deducting the radii of the channels from this (Fig. 3b).

To determine the alignment factor of the collagen fibres, top view images were obtained as follows: 63x objective lens was used with a zoom factor of 1.7 for COL vs MCCG experiments and 4.0 for MCCG studies with different NaCl concentrations, and images were taken at 10 µm-volume stacks with a step size of 4.99 µm. The position of the cover glass was set as *z* = 0 µm, and images were taken from *z* = 10 µm to *z* = 20 µm. Prior to capturing images, the samples were oriented so that the collagen fibres in the field of view of the point of interest were parallel to the x-axis. Two images were taken for each sample, and *n* = 6. For data analysis, a 30 µm × 10 µm window was cropped near the interface between the channel lumen and collagen matrix (Fig. 3c). The cropped image was then converted to 8-bit, and a fast Fourier transform (FFT) image generated from this. The radial sum of the FFT image intensity (*I*) from 0–360° was obtained using the Oval Profile plugin in Image J, and the alignment factor, A_f_, calculated using the formula used in previous studies to determine the orientation of fibrils in gels^19^:

1 $${A}_{f}=\frac{{\int }_{0}^{2\pi }\,I(q,\varphi )\,\cos \,(2\varphi )d\varphi }{{\int }_{0}^{2\pi }\,I(q,\varphi )d\varphi }$$

An A_f_ value of 0 indicates no preference in the direction of fibre alignment, a value between 0 and +1 indicates a preferred alignment in the vertical direction, and a value between −1 and 0 indicates that the fibres preferentially align horizontally in real space.

### Statistical analysis

Two-tailed student’s t-test was used to determine the statistical significance of the differences between COL and MCCG. In comparing MCCGs with different ionic concentrations, one-way ANOVA was first performed on all conditions at different depths, followed by post-hoc Tukey’s test between all pair combinations within the group.

The datasets generated and analysed during the current study are available from the corresponding author on reasonable request.

## Results and Discussion

### Extending Angle and Straightness of Neurite-like Processes in COL and MCCG

Aligned collagen fibres have been shown in numerous studies to guide the growth direction of neural cells, whether they are aligned magnetically^9^, by electrospinning^20^, or using microfluidic devices^7^. In our system, it is thought that the phase separation of collagen solution into a concentrated (collagen matrix) and diluted (lumen) region results in the collagen fibres being aligned in an ordered manner parallel to the interface between these two regions^13^. Thus, we hypothesized that, compared to collagen gel with no collagen fibre alignment (COL), the neurite-like processes of PC12 cells will be guided by the aligned collagen in MCCG.

**Figure 4:**
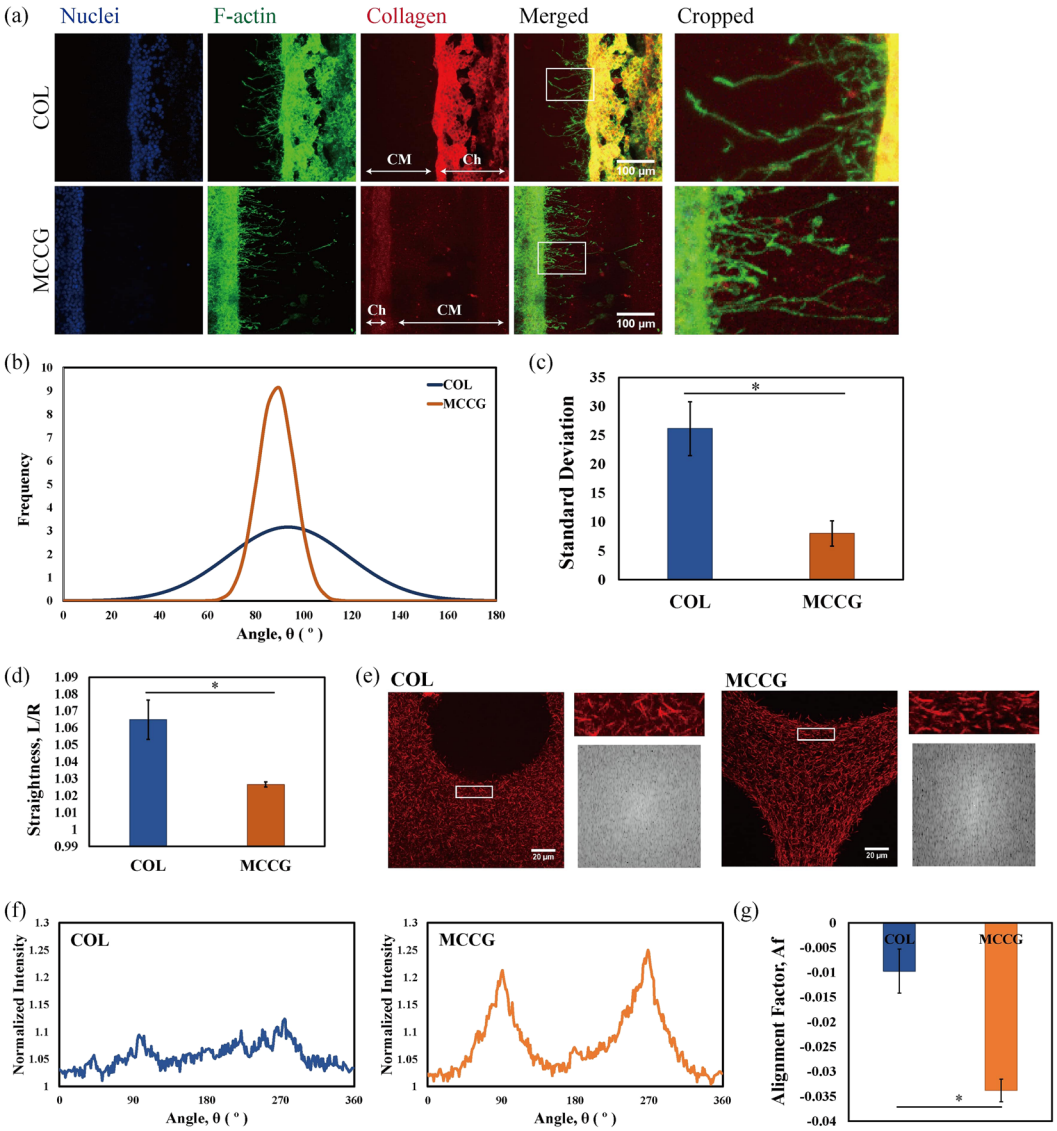
COL vs. MCCG. (**a**) CLSM images of PC12 cells seeded in the channels of COL and MCCG show the neurite-like processes extending into the collagen matrix region. Ch: channel lumen; CM: collagen matrix. (**b**,**c**) The distribution of the angles at which the processes extend relative to the channel structure clearly show that those in MCCG mostly extend perpendicular to the channel, whereas those in COL extend at different angles (*p* = 0.05). (**d**) The processes in MCCG grow significantly straighter than those in COL (*p* = 0.03). (**e**) CRM images of channels without cells and FFT images of the cropped sections show that the collagen fibres in COL are orientated randomly, whereas those in MCCG are aligned in the same direction. (**f**) The relatively uniform peaks in the frequency plot of the FFT intensity of COL indicate that the fibres are randomly aligned, while the two major peaks in that of MCCG indicate that the fibres are highly aligned. (**g**) Alignment factor calculations of the collagen fibres in COL and MCCG show that the degree of alignment of fibres are significantly different (*p* = 0.04). Error bars show standard error of the mean (SEM); *n* = 3. Statistical significance was determined by two-tailed paired student’s t-test, *denotes a significance of *p* < 0.05. Scalebar = (**a**) 100 μm, (**e**) 20 μm.

We observed, from CLSM images of PC12 cells seeded and differentiated in the channels of gels, that the neurite-like processes in MCCG appear to grow in a more ordered manner compared to those in COL (Fig. 4a). We could see that the cells are contained in the channel region (Ch), and extend their processes into the collagen matrix region (CM). From low magnification images of the top view of the MCCG, the neurites were seen to elongate parallel to and along the channel surface when it is close to the surface, whereas from the side view, the neurites appear to extend perpendicular to the channel structure and along the path of collgen fibre alignment (Supplementary Fig. S1). We measured the angles at which the neurite-like processes extend relative to the channel structure (Fig. 4b,c), which clearly confirmed that the processes in MCCG extend into the collagen region in a manner perpendicular to the channel lumen, whereas those in COL extend in a more disorderly and varied manner. Furthermore, the significant difference in the straightness of the processes (Fig. 4d), calculated as the ratio of contour length to end-to-end length from the tip of the growth cone to the channel wall, indicates that the growth pathway of the processes is highly guided in MCCG.

We have previously shown, using polarized light, that the MCCG possesses birefringence property around the lumen of the channels^16^, indicating that the topography around the channel surface is anisotropic. Here, we used confocal reflectance microscopy (CRM) to visualize the collagen fibrils in order to confirm the orientation of fibres in the gels. When we compared top view CRM images which showed the collagen fibres in COL and MCCG (Fig. 4e), we found that the fibres in COL appear to be orientated in a random manner, whereas those in MCCG were mostly aligned in the same direction. We took the normalized radial sums of FFT intensity of the sections cropped from near the interface between the channel lumen and collagen matrix as a measure of collagen fibre alignment, a method also used in previous studies to show differences in fibre alignment^8,21^. The relatively uniform heights of the peaks in the frequency plot of COL show that the fibres are randomly aligned, in contrast to the two peaks in MCCG at about 90° and 270°, which indicates that the fibres in MCCG are highly aligned in one direction (Fig. 4f). The alignment factor (A_f_) formula that was used in a previous study to determine the orientation of fibrils in a gel^19^ was applied here to determine the degree of alignment. For our experiments, the samples were oriented in such a way that the collagen fibres are parallel to the x-axis (Fig. 3c), which means that an alignment factor of 0 indicates there is no preferred alignment in the fibres, whereas a value higher or lower than 0 indicates that the fibres are aligned in the vertical or horizontal direction, respectively, in real space. The results indicated that the collagen fibres in MCCG were significantly more aligned than those in COL, as the A_f_ value for COL was much closer to 0 than that of MCCG (Fig. 4g). Overall, these results appear to support our hypothesis that the highly aligned collagen fibres in MCCG guide the extension of neurites, whereas the randomly aligned fibres in COL do not provide such guidance.

It should be pointed out, however, that the COL and MCCG in these experiments may not be directly comparable as gels without and with collagen fibre alignment, respectively, as there are also differences in their architecture. While the MCCG has a very high density of channel lumen, we were not able to replicate a similar density of channels in COL – only 4 channels were present in COL. In addition, the MCCG itself possesses different characteristics along its depth – the channels increase in size from the top to bottom of the gel, which translates to decreasing channel density from top to bottom. As the topography of the growth substrate is also known to have an influence in the growth direction of neural cells^17^, we wondered if the size and density of channels in MCCG could therefore also have an influence on the guidance of neurite growth by restricting the area of collagen available to the extending neurite.

It was reported in our previous study that the addition of NaCl affects the phase behaviour of collagen solution, resulting in MCCGs with different architecture – increasing the concentration of NaCl led to an increase in channel diameter and a decrease in the number of channels^18^. Thus, we made use of this method to control the structure of MCCG to determine whether the architecture of the MCCG itself is also involved in guiding the growth of neurites.

### Characterisation of MCCGs Prepared with Different NaCl Concentrations

In our previous study, the properties of MCCG structure were reported only for the top and bottom 300 µm of a 1 mm-thick cylindrical gel of 8 mm diameter^18^. As a different setup was used to prepare rectangular gels in this study, we once again characterised the MCCGs to confirm and enable us to make inferences between the structure of the MCCGs and their guidance properties. For this characterisation study, MCCGs with a width of 6 mm and a length of 5 mm was prepared, with gel formation occurring in the direction of the length of the gel. The gels were cut at various points along the length to investigate the characteristics of the gel at different depths, with z = 0 mm defined as an origin of the gel formation, and z = 5 mm as an end point of the gelation.

**Figure 5:**
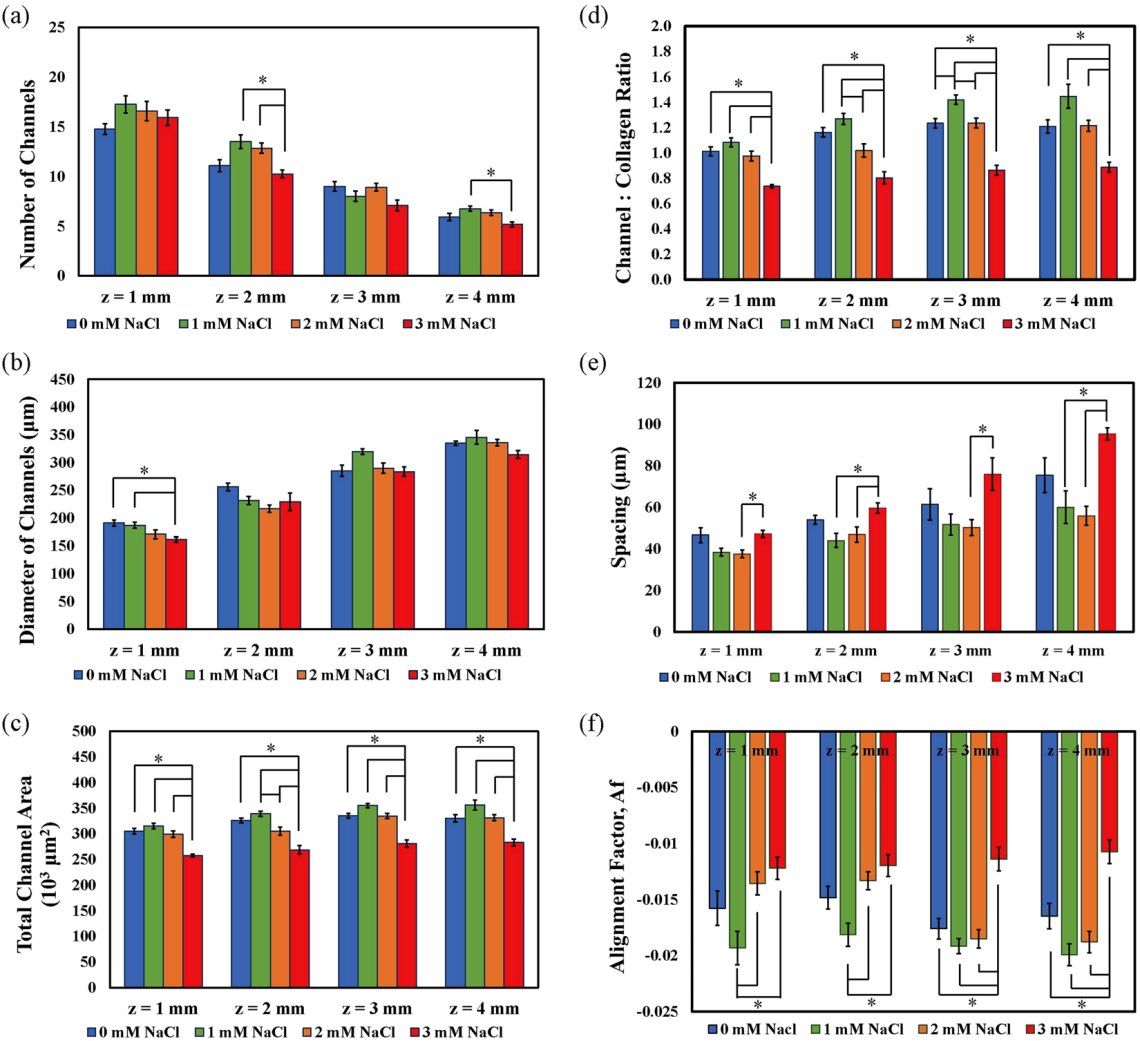
Characterisation of MCCGs prepared with 0 mM, 1 mM, 2 mM, and 3 mM NaCl. (**a**) The number of channels decreased with increasing depth in all conditions, whereas (**b**) the diameter of the channels increased. (**c**) At all depths, the total area occupied by channels was significantly lower in 3 mM NaCl compared to the other conditions, and a similar trend was reflected in (**d**) the density of channels in MCCG which was taken to be the ratio of channel area to collagen matrix area. (**e**) The spacing between adjacent channels were 40–100 µm, and significant were seen between 2 mM and 3 mM NaCl at all depths, and between 1 mM and 3 mM NaCl at z = 2 mm and z = 4 mm. (**f**) All MCCGs had negative alignment factor values, indicating that the collagen fibres are aligned in a preferred direction. At z = 1 mm and z = 2 mm, 1 mM NaCl had significantly higher degree of alignment compared to 2 mM and 3 mM NaCl, whereas at z = 3 mm and z = 4 mm, 3 mM NaCl had a lower degree of alignment compared to all the other conditions. Error bars show standard error of the mean (SEM); *n* = 6. *Denotes a significance of *p* < 0.05 by Tukey’s post hoc test performed between pairs after performing one-way ANOVA on the groups.

From top view CRM images obtained at z = 1 mm, 2 mm, 3 mm, and 4 mm depths of the MCCGs prepared with 0 mM, 1 mM, 2 mM, and 3 mM NaCl (Supplementary Fig. S2), we counted the number of channels, measured the diameter of the channels, and measured the total area occupied by the channels. In all conditions, with increasing depth, the number of channels and diameter of channels decreased and increased, respectively (Fig. 5a,b). This pattern is similar to that reported in the previous study, and also indicates that at increasing depths, these values do not reach a plateau. There were no significant differences in the number of channels between the MCCGs prepared at different NaCl concentrations at z = 1 mm and z = 3 mm, but the difference between 1 mM and 3 mM NaCl at z = 2 mm and z = 4 mm were significant, as was that of 2 mM and 3 mM at z = 2 mm. At z greater than 2 mm, there were no differences in the diameter of channels between the different MCCGs, but at z = 1 mm, the diameters in 0 mM and 1 mM were significantly different to those of 3 mM. Thus, it would appear that, in general, there is not much structural differences between the MCCGs prepared with different NaCl concentrations in terms of the number and size of the channels.

Nevertheless, as all channels including not-whole channels at the edges of the image were counted, but only the diameters of whole channels were measured, these may not fully represent the channel density of the MCCG. Instead, we measured the total area occupied by the channels, and determined the density of channels by the ratio of of the areas occupied by channels to that occupied by the collagen matrix (Fig. 5c,d). At all depths, the density of channels of 3 mM NaCl was significantly lower than those of other NaCl concentrations, whilst a significant difference was observed between 1 mM and 2 mM at z = 2 mm and z = 3 mm, and between 0 mM and 1 mM at z = 3 mm only. The results thus far imply that the channels in 3 mM NaCl MCCG are spread out further from one another, as less space is occupied by channels in 3 mM NaCl despite there being no difference in the diameter of channels between the different conditions. Measuring the distance between adjacent channels (Fig. 5e), it was found that the spacing was significantly higher in 3 mM NaCl at all depths between 2 mM NaCl and 3 mM NaCl, and at z = 2 mm and z = 4 mm between 1 mM NaCl and 3 mM NaCl. These affirmed that the macroscopic structure of MCCG can be controlled by NaCl concentration.

Changing the ionic concentration has an effect on the rate of collagen gel formation; it has been shown that with increased ionic concentration, the lag period before precipitation of the nucleus begins increases, and growth is also slowed down over an increased period^14^. As the phase separation process is pinned by the formation of the collagen network^13^, a slower growth rate of the network allows a period in which the collagen may separate into a concentrated and diluted phase. Thus, it is expected that channels with larger diameters will be formed with increased ionic concentration. However, we did not find that an increase in NaCl concentration led to increased diameter of channels. In contrast, at z = 1 mm, the diameter of 3 mM NaCl MCCG was actually smaller than those of 0 mM and 1 mM NaCl. In addition, the total channel area of 3 mM NaCl was lower than the other conditions. This opposing result could perhaps be due to the spinodal decomposition process by which MCCG is formed. In the unstable system in which spinodal decomposition takes place, molecules flow from a low concentration regions to high concentration regions, and this is the process which gives rise to the diluted and concentrated regions that form the channels and collagen matrix, respectively. It is possible that when the rate of collagen network formation is slowed by increased ionic concentrations, more collagen molecules from the lower regions of the gel flow upward to the more concentrated regions. This may explain the lower channel area and high collagen area, lower channel density, and larger spacing between adjacent channels in 3 mM NaCl.

The lower density of channels due to higher ionic concentration is also expected to have an effect on the alignment of collagen fibres. With a lower area of surfaces, and hence lower surface free energy, the collagen fibres may be less aligned. All samples of MCCG had an alignment factor of negative value, indicating that the majority of collagen fibres are oriented in a preferred direction (Fig. 5f and Supplementary Fig. S3). At z = 1 mm and z = 2 mm, the degree of alignment in 1 mM NaCl was significantly higher than that of 2 mM and 3 mM NaCl, whereas at z = 3 mm and z = 4 mm, the 3 mM NaCl MCCG has significantly lower degree of alignment compared to all other conditions. Thus, as well as having an affect on the macroscopic structure of MCCG, NaCl concentration also affects the degree of alignment of collagen fibres near the channel surface. The difference in A_f_ values between MCCG without the addition of NaCl in the COL vs. MCCG experiment (−0.034 in Fig. 4g) and that in the studies of MCCG with different NaCl concentrations (−0.016 in Fig. 5f) may have been attributed to the difference in zoom factors used (ZF = 1.7 and 4.0, respectively), as this has an effect on the resolution of the obtained images.

We investigated the effects of NaCl concentrations on the structural parameters for the MCCGs prepared at NaCl concentration between 0 and 1 mM. The results are shown in Supplementary Fig. S4. However, the differences in structures for the MCCGs were little. Therefore, the hierarchical structure of MCCG are less effective between 0 and 1 mM.

On the other hand, we could also infer from these results the relationship between the proportion of channel to collagen matrix and the degree of alignment – a high channel:collagen ratio correlates with a higher degree of alignment, and vice versa. This is in line with the thought that the orientation of collagen fibres occurs mainly at the interface between the channel and collagen matrix region^13^. That is, a higher total area of channels translates to a larger total channel-matrix interface where collagen fibres align, resulting in a higher degree of alignment.

### Extending Angle and Straightness of Neurite-like Processes in MCCGs Prepared with Different Ionic Concentrations

**Figure 6:**
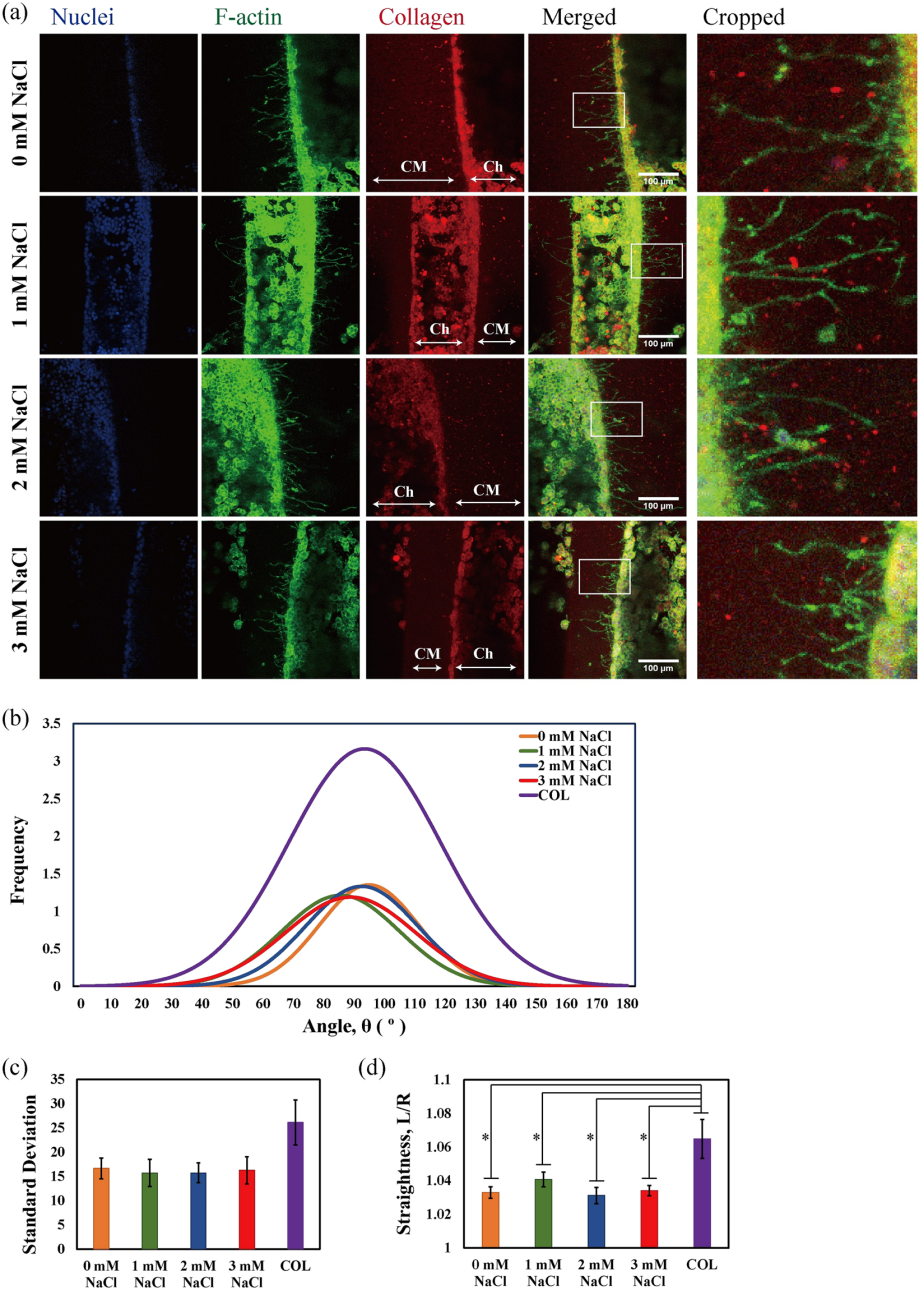
Cells seeded in MCCGs with different ionic concentrations. (**a**) CLSM images of PC12 cells seeded in the channels of MCCGs with different NaCl concentrations. Ch: channel lumen; CM: collagen matrix. (**b**,**c**) There were no significant differences in the angles at which the neurite-like processes of PC12 extended into the collagen matrix regions in all conditions, but (**d**) the growth processes of the neurites were significantly straighter in all MCCGs compared to COL. Error bars show standard error of the mean (SEM); *n* = 6. Statistical significance was determined by Tukey’s post hoc test performed between pairs after performing one-way ANOVA, *p* < 0.05. Scalebar = 100 μm.

Figure 6a shows the CLSM results of the experiments with PC12 repeated in MCCGs prepared with different concentrations of NaCl. Although we found that there exists structural differences in the properties of the MCCGs, no significant differences were found in the angles at which the neurites extend into the collagen matrix and their straightness (Fig. 6b–d). Compared to COL, although the distribution of angles were not significantly different in the different MCCGs, the results indicate that the distributions of angles for MCCGs are narrower than that for MCCG. Furthermore, the neurites in all MCCGs were significantly straighter than those in COL. Therefore, guidance properties for the growth direction of PC12 neurite-like processes of MCCGs were superior to those for COL. However, these results appear to suggest that differences in the structural properties of MCCGs, such as the spacing between channels and the degree of alignment of collagen fibres, do not affect the ability of MCCGs to guide the growth direction of PC12 neurite-like processes.

As a summary of the results of our experiments, we found that MCCG asserts a guiding effect on the growth pathway of the neurite-like processes of PC12 cells compared to COL. However, we also found that significant structural differences in MCCGs prepared with different NaCl concentrations had no obvious effects on the guidance property of MCCG. Nevertheless, an argument can be made for the case of the collagen fibre alignment being the main factor contributing to the guidance property of the MCCG.

The anisotropy of a biomaterial is thought to have an influence on cell behaviour, including the alignment of neuronal cells^22^. Incidentally, although the MCCG appears isotropic macroscopically with its channel structures, the circumferential arrangement of collagen fibres around the channel lumens lends the MCCG an anisotropic feature^13^. Given the significant differences in the growth behaviour of neurite-like processes of PC12 cells seeded in COL (which is isotropic) and MCCG (which is anisotropic), it can thus be said that the guidance property of the MCCG is due to the alignment of collagen fibres around the channels which confers anisotropic properties to the MCCG.

Assuming then, that the alignment factor of collagen fibres correlates with the degree of anisotropy, we could say that 1 mM NaCl MCCG has a higher degree of anisotropy than 3 mM NaCl MCCG. In a study investigating the effect of the degree of anisotropy on neurite contact guidance, it was shown that neurite contact guidance increases with increasing directionality (higher anisotropy), with a threshold effect^23^. By applying our method of measuring A_f_ on the figures of Tonazzini *et al*.^23^, we could extrapolate that the A_f_ values in their study were: approximately 0.011 for 5.5 dB (no neurite guidance), 0.017 for 7.9 dB (threshold value), and 0.046 for 12.2 dB (high neurite guidance); the positive values stem from the vertical direction in which the patterns are oriented, in contrast to the horizontal direction in which collagen fibres are oriented in our studies. Our A_f_ results of lower resolution images where COL = −0.010 and MCCG = −0.034 appears to agree with these values, as we found clearly lower guidance in COL than in MCCG. Furthermore, in the study with MCCGs with different NaCl concentrations where we found neurite guidance in all conditions, the average A_f_ values were: 0 mM = −0.016, 1 mM = −0.019, 2 mM = −0.016 and 3 mM = −0.012. As we found no significant differences in neurite guidance in 1 mM and 3 mM NaCl MCCGs despite significant differences in their alignment factors, it appears that the MCCGs within the experimental range of this study were in the range of the threshold at which increased anisotropy would lead to increased contact guidance. Thus, the threshold effect may also explain why there were no differences in the guidance properties of the various MCCGs.

Although we did not use any chemical cross-linkers to strengthen the mechanical properties of the hydrogels in this study, we did not observe significant contraction of the gels over the culture period. Cells are known to interact with their extracellular matrix and it is likely that they also modify the collagen matrix of the MCCG in which they are seeded. However, the presence of the cells in the channels makes it difficult to accurately analyse the properties of the gel separately, and thus it is difficult to draw conclusions about the relationship between changes in structural properties during prolonged culture and the behaviour of the cells.

## Conclusion

We have shown in this study that the MCCG has the capability to guide the growth pathway of neurites, and that this guidance property is most likely due to the structural anisotropy of MCCG which is contributed by the alignment of collagen fibres. Thus, the MCCG has potential applications as a biomaterial for neural tissue engineering applications, where the ability to control neurite growth is an important feature. Additionally, we have reaffirmed that the structural properties and collagen fibre alignment of MCCG can be altered by increasing ionic strength. Further studies may be required to more fully understand the effects of this on other properties such as mesh size and mechanical properties, but this study shows that we could tailor MCCG to obtain desired properties. In addition, other factors such as pH, temperature, and the size of MCCG may also have an effect on how MCCG forms, further opening a wide array of possibilities with which we can control the properties of MCCG.

## Electronic supplementary material

Supplemental Information
