# Supplementary material for: Anisotropic Multi-channel Collagen Gel (MCCG) Guides the Growth Direction of the Neurite-like Processes of PC12 Cells
Source: Sci Rep. 2018 Sep 17;8:13901. doi: 10.1038/s41598-018-32156-0 (PMC6141479; doi:10.1038/s41598-018-32156-0)
Supplement: Supplementary file 1 — Supplemental Information [file 41598_2018_32156_MOESM1_ESM.pdf]

Supplemental Information for "Anisotropic Multi-channel Collagen Gel (MCCG) Guides the Growth Direction of the Neurite-like Processes of PC12 Cells"

Isabel Koh[a], Kazuya Furusawa[b,c,d*], Hisashi Haga[b,c]

[a]Graduate School of Life Science, Hokkaido University, [b]Faculty of Advanced Life Science, Hokkaido University, [c]Global Station for Soft Matter, Global Institution for Collaborative Research and Education, Hokkaido University, [d]Department of Environmental and Food Sciences, Fukui University of Technology.

Supplementary Information

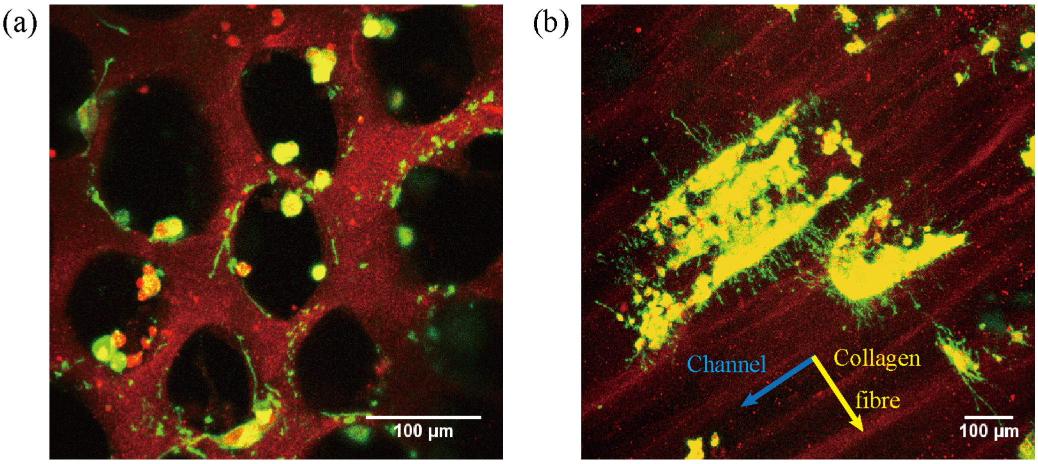

Supplementary Figure S1. Low magnification images of the MCCG seeded with PC12 cells show that (a) from the top view, the neurites are seen to extend parallel to and along the channel surface when it is close to the surface, whereas (b) from the side view, the neurites appear to extend perpendicular to the channel structure and along the alignment of collagen fibre. The blue and yellow arros in (b) indicate the direction of the channel structure and collagen fibre, respectively. Scalebar = 100 µm.

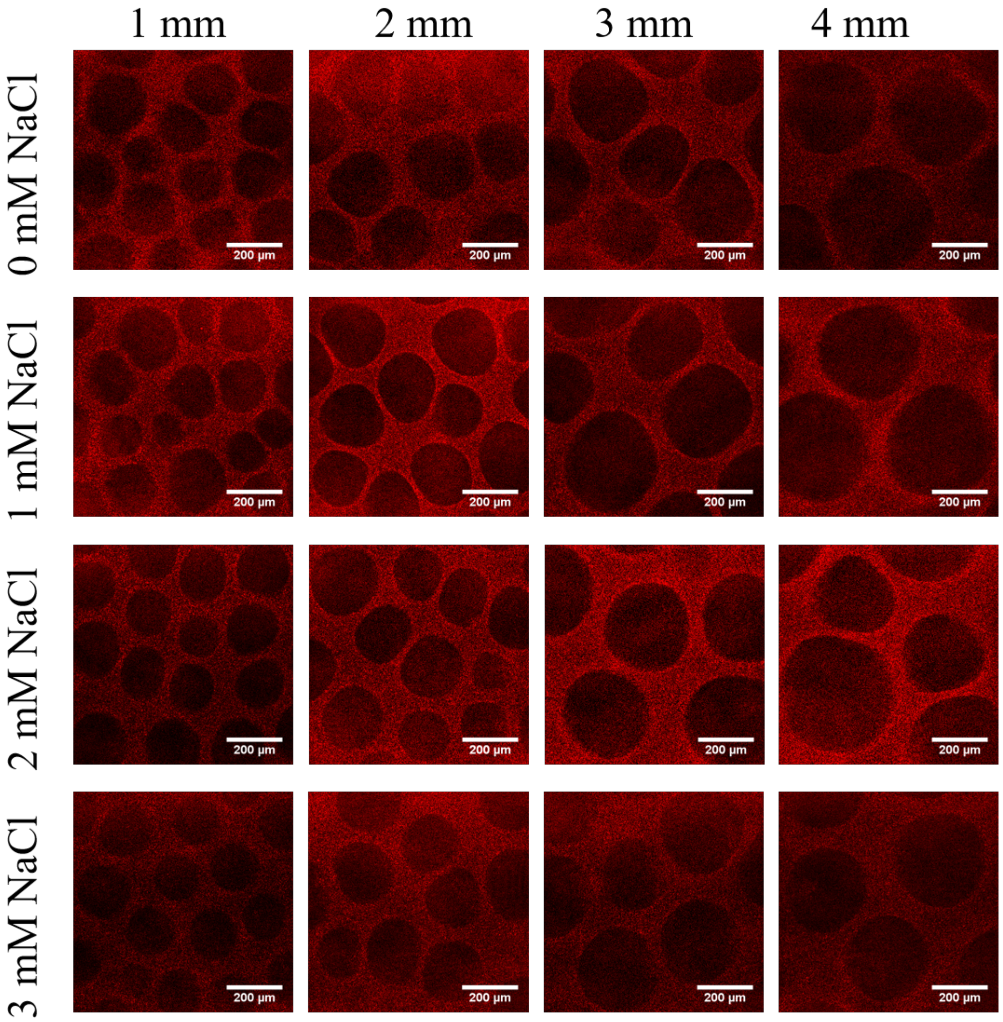

Supplementary Figure S2. Characterisation of MCCGs prepared with 0 mM, 1 mM, 2 mM, and 3 mM NaCl. Top-view CRM images taken with 5x objective show the channel structure. Scalebar = 200 µm.

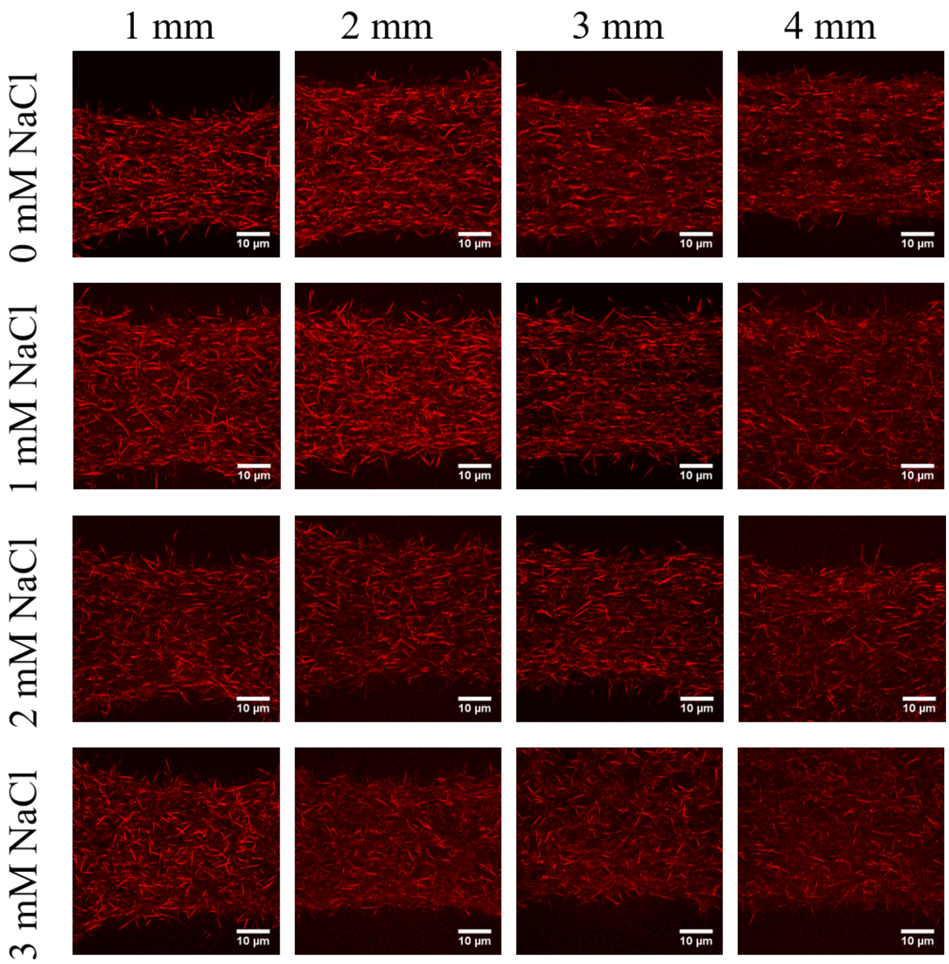

Supplementary Figure S3. Characterisation of MCCGs prepared with 0 mM, 1 mM, 2 mM, and 3 mM NaCl. Top-view CRM images taken with 64x objective show the collagen fibres. Scalebar = 10 µm.

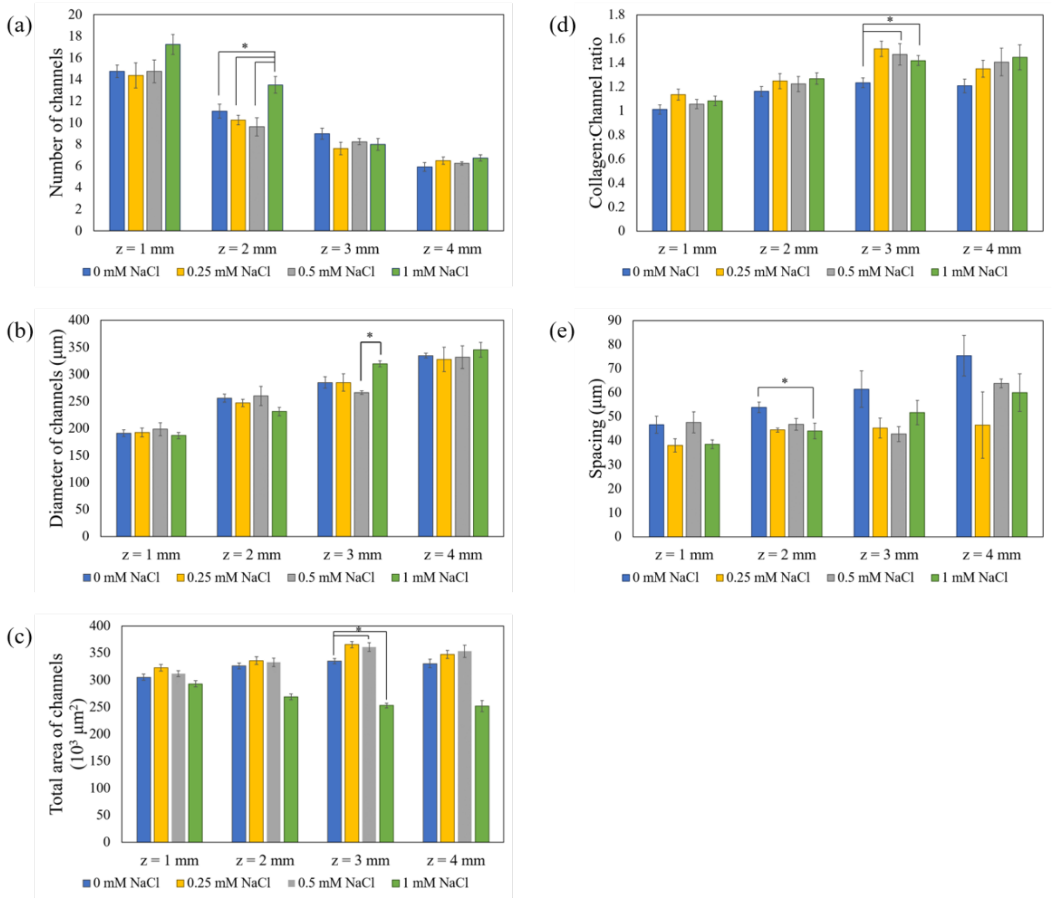

Supplementary Figure S4. (a) Number of channels, (b) diameter of channels, (c) total area of channels, (d) collagen:channel ratio, and (e) spacing between adjacent channels of MCCGs prepared with 0 mM, 0.25 mM, 0.50 mM, and 1 mM NaCl. Error bars show standard error of the mean (SEM). Statistical significance was determined by Tukey's post hoc test performed between pairs after one-way ANOVA, $p<0.05$.
